# GSTM1 and GSTT1 null genotype increase the risk of hepatocellular carcinoma: evidence based on 46 studies

**DOI:** 10.1186/s12935-019-0792-3

**Published:** 2019-03-29

**Authors:** Shanli Li, Feng Xue, Yi Zheng, Pengtao Yang, Shuai Lin, Yujiao Deng, Peng Xu, Linghui Zhou, Qian Hao, Zhen Zhai, Ying Wu, Zhijun Dai, Shu Chen

**Affiliations:** 1 Department of Interventional Vascular Surgery, The Affiliated Bao ji Central Hospital of Xi’an Jiaotong University College of Medicine, Bao ji, 721008 Shaan xi China; 2 Department of Oncology, The Second Affiliated Hospital of Xi’an Jiaotong University, Xi’an, 710004 China; 3 Department of Hepatobiliary Surgery, The 3rd Affiliated Teaching Hospital of Xinjiang Medical University (Affiliated Tumor Hospital), Urumqi, 830000 China

**Keywords:** Glutathione *S*-transferase, Hepatocellular carcinoma, GSTM1, GSTT1, Meta-analysis

## Abstract

**Background:**

It is well known that hepatocellular carcinoma (HCC) has been one of the most life-threatening diseases all over the world. Plenty of internal and extrinsic factors have been proven to be related to HCC, such as gene mutation, viral hepatitis, and Nitrosamines. Though previous studies demonstrated that glutathione *S*-transferase (GST) genotypes are associated with HCC, the conclusions are inconsistent. Therefore, we carried on a renewed meta-analysis to expound the connection between the null GSTM1, GSTT1 polymorphisms and the risk of HCC.

**Methods:**

We searched PubMed, Web of Science, Embase, and CNKI databases to select qualified researches which satisfied the inclusion criteria up to July 31, 2018. Finally, we selected 41 articles with 6124 cases and 9781 controls in this meta-analysis. We use ORs and 95% confidence interval (CI) to evaluate the correlation intension between the GSTM1 and GSTT1 null genes and the risk of HCC. All the statistical processes were executed by Stata (version 12.0).

**Results:**

The pooled analysis showed that both GSTM1 null genotypes (OR = 1.37, 95% CI = 1.18–1.59) and GSTT1 null genotypes (OR = 1.43, 95% CI = 1.23–1.66) increased the risk of HCC. And GSTM1–GSTT1 dual-null genotypes also increased the risk of HCC (OR = 1.58, 95% CI = 1.22–2.05). In the subgroup analysis, we obtained significant results among Asians when stratified by race, and the results are GSTM1 null OR = 1.44, 95% CI = (1.22–1.71), GSTT1 null OR = 1.48, 95% CI = (1.25–1.77), GSTM1–GSTT1 null OR = 1.58, 95% CI = (1.19–2.09), while we didn’t obtain significant results among Caucasians or Africans. Stratified analyses on the type of control indicated a higher risk of HCC associated with GSTM1, GSTT1 single null genotypes and GSTM1–GSTT1 dual-null genotypes in healthy people. No evidence of significant connection was discovered in chronic liver disease (CLD) except in GSTT1 single null.

**Conclusions:**

Our study indicated that an individual who carries the GSTM1, GSTT1 single null genotypes and GSTT1–GSTM1 dual-null genotypes is more likely to develop HCC.

**Electronic supplementary material:**

The online version of this article (10.1186/s12935-019-0792-3) contains supplementary material, which is available to authorized users.

## Background

Hepatocellular carcinoma (HCC) has been one of the most common and terrifying cancer on account of a high mortality, which is turning into the third major cancer all over the world and is the second important factor of cancer-related deaths in China [1, 2]. It is evaluated that 70% to 90% liver cancers in the world are hepatocellular carcinoma. The pathogenesis of HCC is extremely complicated which including the internal and external factors, in other words, environmental conditions and individual genetic background both matters. Among those multiple risk factors, chronic infection with hepatitis B virus (HBV) has a strong relationship with the HCC prevalence. The high incidence rate of hepatocellular carcinoma in a certain district of Asia and Africa is prevailingly because of the high infection rate of chronic HBV, which follows the development of the path from hepatitis to liver cirrhosis and turns into HCC at last [3]. Besides, heavy consumption of alcohol and tobacco and family history of related tumor have been certified to have an intimate connection with a higher risk of HCC by numerous epidemiological and statistical researches [4–7].

When we talk about the internal factors, it is inevitable to study the effect of changes in genetic expression of certain genes or cytokines. The change of many signaling pathways and its molecules such as extracellular regulated protein kinases, antichymotrypsin, growth factor receptor-bound protein 2 (Grb2)—associated binding protein 2 have been found to be related to the blossom of HCC [8]. Among those expected genes, the GSTs have been a hot topic for its tremendous impact [9]. The GSTs play a crucial role in phase II isoenzymes which have tremendous defensive function because of its detoxication and regulating effect. Therefore, the decline of content of GSTs is highly possible to bring out carcinogenesis.

Among all the GSTs, the GSTM1 and GSTT1 have been extensively found related to various types of cancers [10]. Though former studies recognized the great value of GSTM1 and GSTT1 null genotypes, they did not meet a widely-agreed conclusion which could be generated by the limitation of sample size, statistical methods and other unsuspected reasons. For instance, certain studies included less than a few hundred HCC cases could lead to a conclusion which lacks credibility. To eliminate this interference factors in a better way, we therefore carried on a renewed meta-analysis to comprehensively evaluate the total harm of GSTM1 and GSTT1 null genotypes on the progress of HCC.

## Methods

### Identification and selection of relevant studies

We sought several databases including but not limited to PubMed, Web of Science, Embase, and CNKI for correlative researches up to July 31, 2018 for using various combination of those keywords: GSTM1, GSTT1, hepatocellular carcinoma, polymorphism and HCC.

### Inclusion and exclusion criteria

We adopted the publications which assess the correlation of the GSTM1 and GSTT1 polymorphisms with HCC. Qualified studies must satisfy each requirement which was listed below: (1) must be a case–control study or a cohort study (2) have primitive and firsthand data and be independent from other researches, (3) have complete data to conduct statistical analysis.

Exclusion criteria are: (1) primitive and firsthand data are not available; (2) we excluded the studies which were based upon the same subjects and published by the same authors and selected the biggest samples or the latest data; (3) Additionally, meeting abstract, case reports, and other meta-analysis were not adopted.

### Data extraction

Two participants independently extracted the data that we need cautiously from all qualified publications, and the related information included the first author, race of the samples, source of control, year of publication, respective number of each case and control, genotyping method, and type of control. Any potential inconsistency of the results was discussed and settled by consensus.

### Statistical analysis

For GSTT1 and GSTM1, we respectively compared null genotype and normal genotype frequencies between cases and controls. And we calculated its ORs and 95% confidence interval (CI) to evaluate the correlation intension between the GSTM1 and GSTT1 null genes and the risk of HCC. Cochran’s Q statistic was used to assess heterogeneity and the I^2^ statistic to measure the inconsistency [11–13]. Subgroup analyses were executed by ethnicity (Asians, Africans, Caucasians), and type of control (healthy and chronic liver disease). We made Begg’s funnel plot to check whether there exists the potential publication bias. In order to measure the steadiness and reliability of our results, we carried out the sensitivity analyses by removing each single study and reanalyzing the rest to check the potential biases [14, 15]. All the statistical processes were executed by Stata (version 12.0). The *P* values were two-sided, and the *P* < 0.05 was taken for statistically significant. In addition, for GSTT1–GSTM1 both null, we use the same method to analyze the datum.

## Results

### Study characteristics

**Fig. 1:**
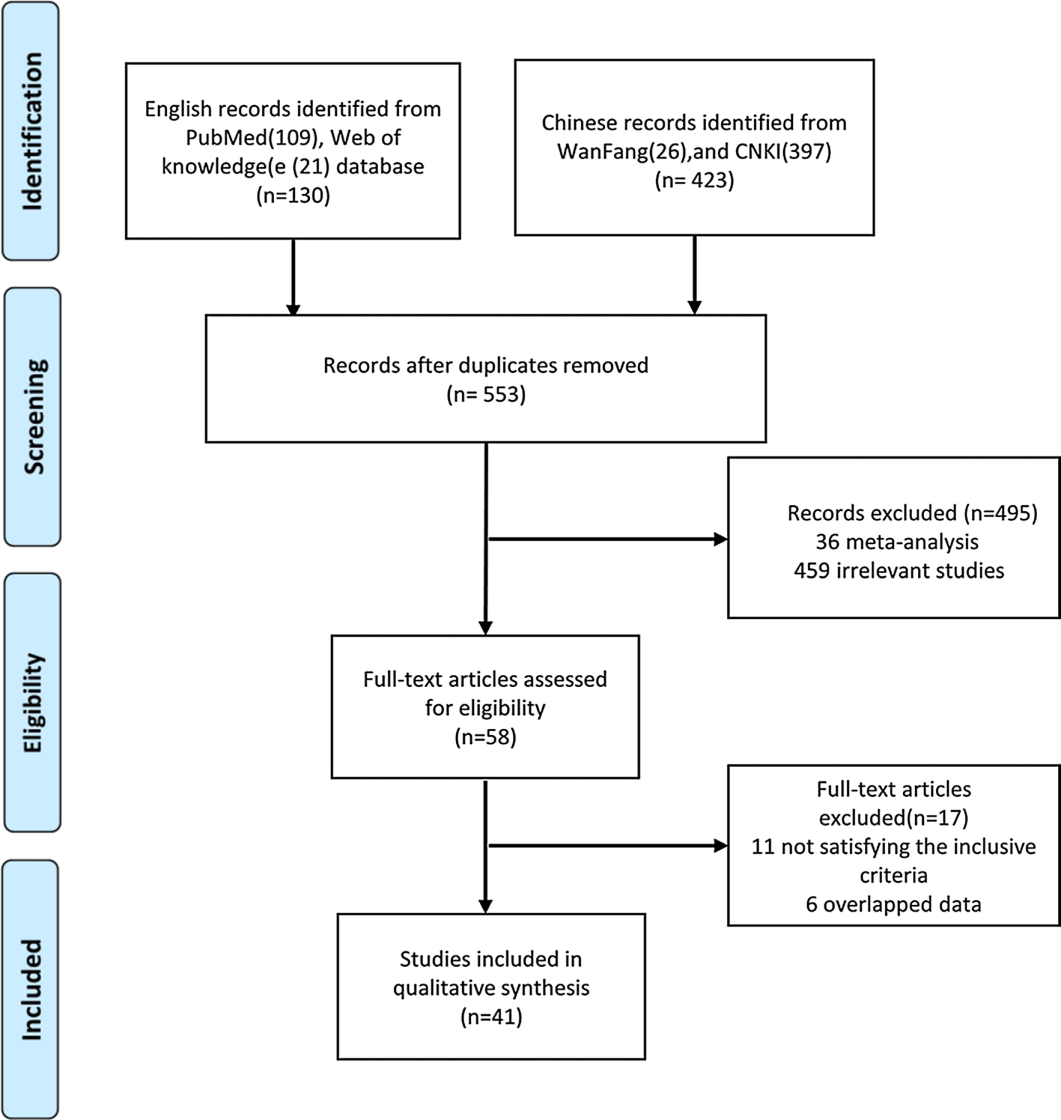
The flow diagram of the meta-analysis, according to the PRISMA 2009. CNKI: China National Knowledge Infrastructure

We have selected 553 correlative papers in term of the keywords from the databases, and 512 articles were removed for not meeting all the inclusion criteria. A total of 41 qualified papers were adopted in the end and all the studies obtained high-quality score according to the Newcastle–Ottawa Scale (NOS) (Table 1, Additional file 1: Table S1). Some articles included two groups in terms of a different type of control or source of control. We divided them into two studies to analyze them conveniently [16–20]. As a result, we got 46 studies and all the studies with 15905 objects for GSTM1 and 34 of them with 12589 objects for GSTT1, respectively [16–56]. The process of the selection of the literature was shown in Fig. 1. Thirty nine was involved in Asian, 4 was involved in Caucasian, 3 was involved in African among these studies. We exhibited the main characteristics of the studies and the main results in Tables 1 and 2, severally (more detailed information was showed in Additional file 1: Table S2).

**Table 1:** Characteristics of the studies included in the meta-analysis

Study	Year	Ethnicity	Source of control	No. of cases/controls	Genotyping method	Type of control	Study quality
Boccia [21]	2015	Caucasian	Hospital	221/290	RFLP-PCR	Healthy	6
Chen [22]	2012	Asian	Population	76/68	Multiplex PCR	Healthy	8
Tang [23]	2012	Asian	Population	150/150	RFLP-PCR	Healthy	7
Li [24]	2012	Asian	Population	476/481	PCR-CTPP	Healthy	7
Sarma [25]	2012	Asian	Hospital	68/123	PCR	CLD	8
Xiao [26]	2011	Asian	Hospital	210/75	PCR	Healthy	8
Wei [27]	2010	Asian	Population	181/641	PCR	Healthy	7
Kao [28]	2010	Asian	Population	102/386	PCR	Healthy	8
Asim [29]	2010	Asian	Hospital	254/525	Multiplex PCR	CLD	8
Yang [30]	2009	Asian	Population	100/60	Multiplex PCR	Healthy	6
Takeshi [16]	2009	Asian	Hospital	209/256	RFLP-PCR	Healthy	8
Takeshi [16]	2009	Asian	Hospital	209/381	RFLP-PCR	CLD	8
He [31]	2008	Asian	Population	105/151	Multiplex PCR	Healthy	6
Kiran [17]	2008	Asian	Hospital	63/169	RFLP-PCR	Healthy	6
Kiran [17]	2008	Asian	Hospital	63/174	RFLP-PCR	CLD	6
Elhamy [32]	2008	African	Hospital	60/50	PCR	CLD	7
Borentain [33]	2007	Caucasian	Population	56/89	ARMS-PCR	Healthy	7
Deng [34]	2006	Asian	Population	88/115	Multiplex PCR	Healthy	6
Long [35]	2006	Asian	Hospital	257/649	RFLP-PCR	CLD	7
Ladero [36]	2006	Caucasian	Population	184/329	Multiplex PCR	Healthy	7
Zhang [18]	2005	Asian	Population	60/73	Multiplex PCR	Healthy	6
Zhang [18]	2005	Asian	Hospital	60/66	Multiplex PCR	CLD	6
Guo [37]	2005	Asian	Population	95/103	Multiplex PCR	Healthy	8
Long [38]	2005	Asian	Population	140/536	PCR	Healthy	8
Deng [19]	2005	Asian	Population	181/360	PCR	Healthy	7
Deng [19]	2005	Asian	Population	110/135	PCR	Healthy	7
He [39]	2005	Asian	Population	105/151	Multiplex PCR	Healthy	8
Gelatti [40]	2005	Caucasian	Hospital	200/400	RFLP-PCR	Healthy	7
Kirk [41]	2005	African	Hospital	149/294	Multiplex PCR	Healthy	7
Ma [42]	2005	Asian	Population	62/73	PCR	CLD	6
Zhu [20]	2005	Asian	Population	91/130	Multiplex PCR	Healthy	7
Zhu [20]	2005	Asian	Hospital	91/118	Multiplex PCR	CLD	7
Li [43]	2004	Asian	Population	207/207	Multiplex PCR	Healthy	8
Yu [44]	2003	Asian	Hospital	577/389	RFLP-PCR	CLD	7
Liu [45]	2003	Asian	Population	51/53	RFLP-PCR	Healthy	7
Munaka [46]	2003	Asian	Hospital	78/138	Multiplex PCR	Healthy	6
Chen [47]	2002	Asian	Hospital	93/35	PCR	Healthy	6
Edine [48]	2001	African	Population	112/194	Multiplex PCR	Healthy	6
Sun [49]	2001	Asian	Hospital	79/149	PCR	CLD	8
Zhu [50]	2001	Asian	Population	52/100	Multiplex PCR	Healthy	8
Bian [51]	2000	Asian	Hospital	63/88	Multiplex PCR	Healthy	8
Wu [52]	2000	Asian	Population	54/136	Multiplex PCR	Healthy	7
Yu [53]	1999	Asian	Population	88/375	PCR	CLD	7
Dong [54]	1997	Asian	Population	54/54	PCR	Healthy	8
Dong [55]	1997	Asian	Population	110/112	PCR	Healthy	8
Yu [56]	1995	Asian	Hospital	30/150	PCR	CLD	6

*PCR* polymerase chain reaction, *RFLP* restriction fragment length polymorphism, *ARMS* the amplification refractory mutation system, *PCR-CTPP* polymerase chain reaction with confronting two-pair primers, *CLD* chronic liver disease

**Table 2:** Main results of the meta-analysis on the association between GSTM1 and GSTT1 null polymorphisms and HCC risk

Null genotype	No. of studies	OR (95% CI)	P(Q)	I^2^ (%)	P(H)
GSTM1	46	*1.37 (1.18–1.59)*	< 0.001	77.2	< 0.001
Subgroup analysis					
Ethnicity					
Asian	39	*1.44 (1.22–1.71)*	< 0.001	78.2	< 0.001
African	3	1.31 (0.98–1.76)	0.398	0	0.073
Caucasian	4	0.93 (0.77–1.13)	0.312	16.0	0.476
Type of control					
Healthy	33	*1.37 (1.17–1.59)*	< 0.001	68.0	< 0.001
CLD	13	1.36 (0.94–1.95)	< 0.001	87.3	0.099
GSTT1	34	*1.43 (1.23–1.66)*	< 0.001	69.3	< 0.001
Subgroup analysis					
Ethnicity					
Asian	28	*1.48 (1.25–1.77)*	< 0.001	71.9	< 0.001
African	3	1.21 (0.77–1.90)	0.122	52.4	0.404
Caucasian	3	1.19 (0.93–1.51)	0.273	23.0	0.164
Type of control					
Healthy	24	*1.42 (1.21–1.66)*	< 0.001	58.2	< 0.001
CLD	10	*1.45 (1.02–2.08)*	< 0.001	82.8	0.041
GSTM1–GSTT1	19	*1.58 (1.22–2.05)*	< 0.001	46.1	0.001
Subgroup analysis					
Ethnicity					
Asian	17	*1.58 (1.19–2.09)*	< 0.001	64.1	0.002
African	1	–	–	–	–
Caucasian	1	–	–	–	–
Type of control					
Healthy	14	*1.61 (1.17–2.22)*	< 0.001	64.7	0.003
CLD	5	1.20 (0.94–1.53)	0.179	36.3	0.153

*GSTM1* glutathione *S*-transferase M1, *GSTT1* glutathione *S*-transferase T1, *CI* confidence interval, *OR* odds ratio, *CLD* chronic liver disease, *OR* odds ratio*, P(Q)* P value of Q test; *P(H)* P value of heterogeneity

### Correlation of GSTM1, GSTT1 single null genotype and GSTM1–GSTT1 double null polymorphism with HCC risk

**Fig. 2:**
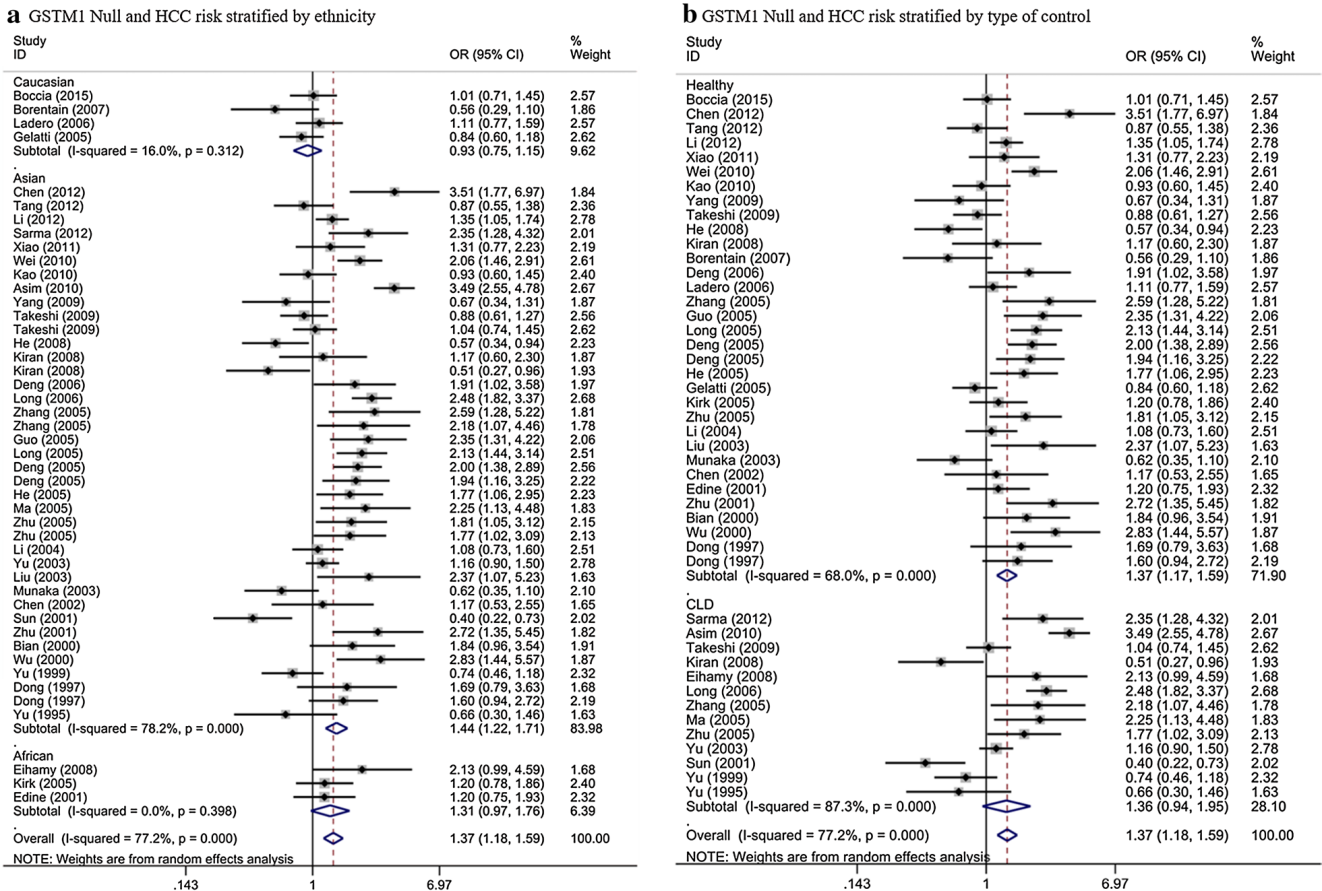
Stratified analysis for the association between GSTM1 null genotype with hepatocellular carcinoma risk (**a** stratified analysis by ethnicity; **b** stratified analysis by type of control). *CI* confidence interval, *OR* odds ratio

In the conjoint analysis, we found an obvious association between the null genotype of GSTM1 and the HCC risk (OR = 1.37, 95% CI = 1.18–1.59, *P* < 0.001, P_heterogeneity_ < 10^−3^). When we conducted a subgroup analysis in term of race, we acquired the similar results in Asian (OR = 1.44, 95% CI = 1.22–1.71, *P* < 0.001, P_heterogeneity_ < 10^−3^) (Fig. 2a). However, we found no significant estimates on Caucasian (OR = 0.93, 95% CI = 0.77–1.13, *P* = 0.476, P_heterogeneity_ = 0.312) and African (OR = 1.31, 95% CI = 0.98–1.76, *P *= 0.073, P_heterogeneity_ = 0.398) populations (Fig. 2a, Table 2). Subgroup analyses in term of type of control showed a valuable result for healthy controls (OR = 1.37, 95% CI = 1.17–1.59, *P* < 0.001, P_heterogeneity_ < 10^−3^), but for CLD controls, we did not find any positive results (OR = 1.36, 95% CI = 0.94–1.95, *P* = 0.099, P_heterogeneity_ < 10^−3^) (Fig. 2b, Table 2).

**Fig. 3:**
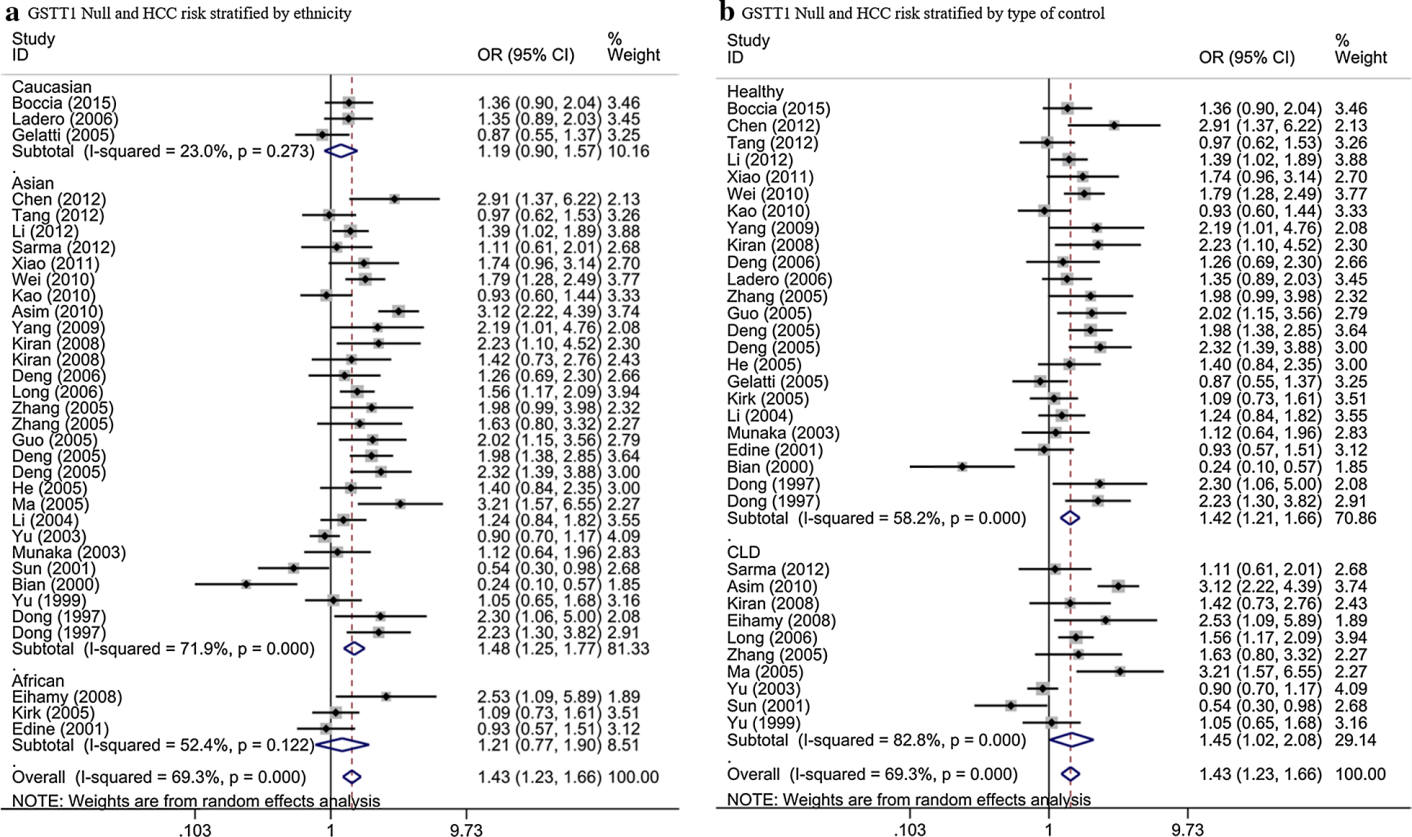
Stratified analysis for the association between GSTT1 null genotype with hepatocellular carcinoma risk (**a** stratified analysis by ethnicity; **b** stratified analysis by type of control). *CI* confidence interval, *OR* odds ratio

The results of 34 studies showed that GSTT1 null genotype increased the risk of HCC (OR = 1.43, 95% CI = 1.23–1.66, *P* < 0.001, P_heterogeneity_ < 10^−3^). When stratified by race, positive results were discovered among Asians (OR = 1.48, 95% CI = 1.25–1.77, *P* < 0.001, P_heterogeneity_ < 10^−3^) (Fig. 3a). Nevertheless, no connections were discovered among Caucasian (OR = 1.19, 95% CI = 0.93–1.51, *P* = 0.164, P_heterogeneity_ = 0.273) and African (OR = 1.21, 95% CI = 0.77–1.90, *P *= 0.404, P_heterogeneity_ = 0.122) (Fig. 3a). In the subgroup analysis based on type of control, we obtained positive results in healthy objects (OR = 1.42, 95% CI = 1.21–1.66, *P* < 0.001, P_heterogeneity_ < 10^−3^). In addition, we got similar conclusion in CLD controls (OR = 1.45, 95% CI = 1.02–2.08, *P* = 0.041, P_heterogeneity_ < 10^−3^) (Fig. 3b; Table 2).

**Fig. 4:**
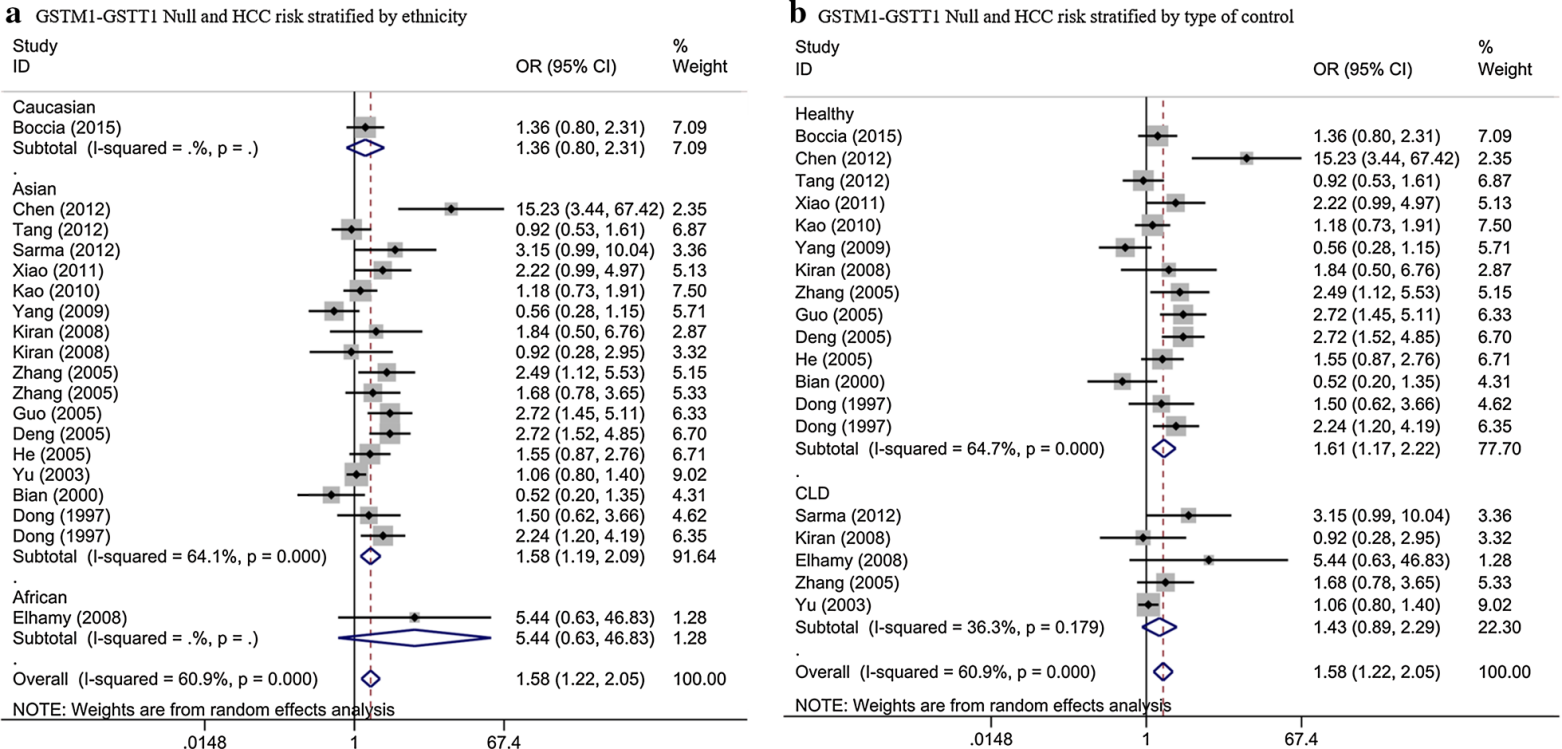
Stratified analysis for the association between GSTM1 and GSTT1 double null genotype with hepatocellular carcinoma risk (**a** stratified analysis by ethnicity; **b** stratified analysis by type of control). *CI* confidence interval, *OR* odds ratio

19 studies with 5063 subjects exhibited the connection between GSTM1 and GSTT1 double null and the risk of HCC. The result showed that GSTM1–GSTT1 double null genotype was related with enhanced risk of HCC (OR = 1.58, 95% CI = 1.22–2.05, *P* = 0.001, P_heterogeneity_ < 10^−3^). When stratified by race, positive results were found among Asians (OR = 1.58, 95% CI = 1.19–2.09, *P *= 0.002, P_heterogeneity_ < 10^−3^) (Fig. 4a) while no associations were found among African and Caucasian (Fig. 4a). When stratified by type of control, significant associations were found in healthy objects (OR = 1.61, 95% CI = 1.17–2.22, *P* = 0.003, P_heterogeneity_ < 10^−3^) (Fig. 4b). But for CLD controls, we did not find positive results (OR = 1.20, 95% CI = 0.94–1.53, *P* = 0.153, P_heterogeneity_ = 0.179) (Fig. 4b, Table 2).

### Publication bias and sensitivity analysis

**Fig. 5:**
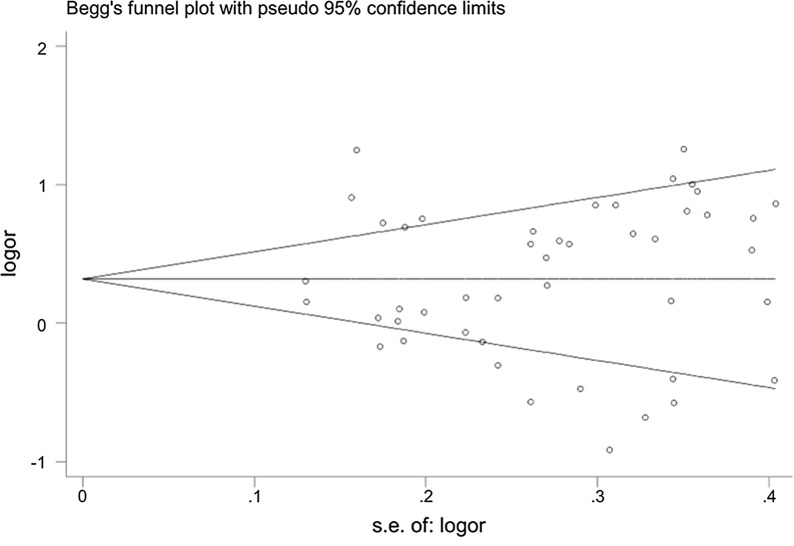
Funnel plots of publication bias for GSTM1 null genotype

**Fig. 6:**
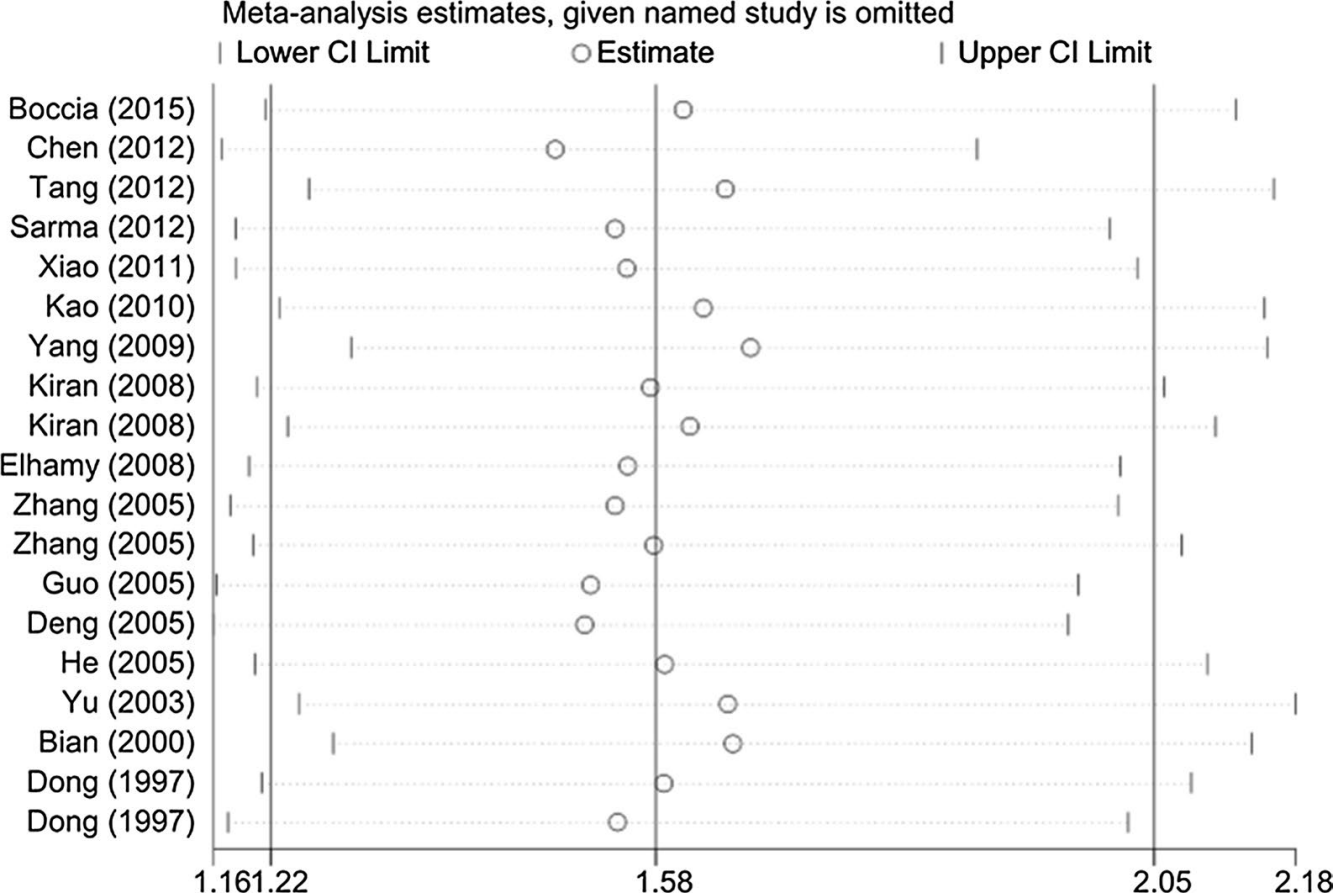
Sensitivity analysis of the associations between GSTM1 and GSTT1 double null genotype with hepatocellular carcinoma risk

We utilized Begg’s and Egger’s test to detect the possible sources of bias. The funnel plots were symmetrical as showed in Fig. 5, indicating that there were no publication bias (*P *> 0.05). Sensitivity analysis was performed to measure the stability of the conclusive results, and we tell that there was no substantial transformation by removing any data set according to sensitivity analysis (Fig. 6). There were no small study effects among the adopted studies showed by Egger’s test.

## Discussion

Previous studies already showed that the null GSTM1 and GSTT1 genotypes were likely to have an association with higher risk of a different type of cancers. The study by Wang zhao-yang et al. reported the null GSTT1 and null GSTM1 might have connection with higher death risk in gastric cancer [57]. Vesna M. Coric’s research showed that the carriers with GSTM1 null genotype is more likely to get renal cell carcinoma [58]. Neha Sharmaa et al. reported that the drop content of GST genotypes seemed to have close relationship with lung cancer and its histological subtypes [59]. There are also many studies focused on the relationship of GSTM1 and GSTT1 null genotypes and HCC risk, however, they did not meet a widely-agreed conclusion. Donna L. White’s study showed that the GSTM1 null and GSTT1 null genotype nearly had no connection with HCC [60]. Kang Song’s research showed that GSTM1 and GSTT1 null genotype may slightly increase the risk of HCC [61]. Ying-Hao Shen’s research found that the GSTM1 and GSTT1 null genotype was associated with an increased risk of HCC [62]. We therefore carried on this renewed meta-analysis to comprehensively evaluate the total risk of HCC when there exists GSTM1 and GSTT1 null genotypes. Our study includes 46 studies and 15905 objects in total compared to previous studies. Furthermore, we evaluated the risk of GSTM1–GSTT1 double null in HCC. Our results showed that the deficient of GSTM1 and GSTT1 genotype may be connected with a higher risk of HCC for its results is statistically significant. Compared to single null of GSTM1 and GSTT1 genotype, we furthermore discovered that individuals with dual genotypes of GSTs genes may be easier to develop HCC, but it still needs further exploration. The number of patients with hepatocellular carcinoma is large, and the diagnosis is late, also the prognosis is poor. These significant results may prompt us to conduct genetic testing on high-risk patients with hepatocellular carcinoma as early as possible, which plays an important role in early diagnosis, improvement of prognosis and reduction of the economic burden.

In the subgroup analysis in term of ethnicity, HCC risk was increased in Asians for single null of GSTM1 and GSTT1 and GSTM1–GSTT1 double-null genotype. However, we could not get the similar conclusion in Caucasians or Africans. This may be affected by diverse living surroundings and different inherited backgrounds. There may exist many other polymorphisms with combined effects that affect cancer susceptibility. As a consequence, the effect of certain individual polymorphism on the risk of cancer could be weakened. Our data also showed that the respectively null genotypes of GSTM1 and GSTT1 genotypes stand a good chance of having a duplicate effect on the HCC. When stratified by ethnicity, significant connection was found in Asian for GSTM1, but not in African and Caucasian. This could be explained by the potential possibility that the African and Caucasian may have certain unknown repair mechanism to avoid the damage caused by deficiency of the GSTM1 gene. It is said that the infection of HBV can easily evolve to liver cirrhosis and finally to HCC [63]. In addition, foodstuffs polluted with aflatoxin B1 (AFB1) are also a threat to livers. AFB1 is a kind of hepatotoxic mycotoxin. It could be produced by fungi of the Aspergillus species when the grains were stored in moist conditions. Once ingested, AFB1 was turned into an active intermediate, then produce certain a substance which may damage DNA [64]. Evidence from literature implies that the GSTs play an important role in the second metabolism phase of AFB1. Kimet al. reported that subjects which carried the null genotype of GSTT1 seem to increase the possibility of occurrence of chromosomal aberrations [65].

Despite our great efforts to check the association between the GSTs genes and the risk of HCC, we must pay attention to some limitations. Firstly, our analysis is a kind of cursory estimate since we had no chance to analyze each individual data. Secondly, the selected trials differ in certain aspects, including differences in trial populations and other potential diseases, and it is possible that methodological differences might have confused the conclusions of subgroups of trials. Thirdly, by including studies published a long time may affect the authenticity of the result.

## Conclusions

All in all, our study showed that the single null of GSTM1, GSTT1 and GSTM1–GSTT1 double null genotypes are all connected with a higher risk of HCC. It is noteworthy that our data revealed that individuals with double-null genotypes of GSTs genes may be more likely to develop into HCC which worth further exploration. Last but not least, we need larger sample epidemiological studies of high quality to make current conclusions more reliable and stable.

## Additional files

**Additional file 1: Table S1.** The evaluation of the data quality in this meta-analysis.

**Additional file 2: Table S2.** The detailed characteristics of the included studies in this meta-analysis.

## Abbreviations

GSTM1: glutathione *S*-transferase M1
GSTT1: glutathione *S*-transferase T1
CI: confidence interval
OR: odds ratio
CLD: chronic liver disease
PCR: polymerase chain reaction
RFLP: restriction fragment length polymorphism
ARMS: the amplification refractory mutation system
PCR-CTPP: polymerase chain reaction with confronting two-pair primers
